# Acknowledgement to Reviewers of *Micromachines* in 2019

**DOI:** 10.3390/mi11010111

**Published:** 2020-01-20

**Authors:** Micromachines Editorial Office

**Affiliations:** MDPI, St. Alban-Anlage 66, 4052 Basel, Switzerland

The editorial team greatly appreciates the reviewers who have dedicated their considerable time and expertise to the journal’s rigorous editorial process over the past 12 months, regardless of whether the papers are finally published or not. In 2019, a total of 885 papers were published in the journal, with a median time to first decision of 13 days and a median time from submission to publication of 34 days. The editors would like to express their sincere gratitude to the following reviewers for their generous contribution in 2019:
Abbasi, Qammer H.Liu, ManingAbdel-Motaleb, Ibrahim M.Liu, RanAbdolvand, RezaLiu, SongtaoAbe, EisukeLiu, TianyuAbedini Nassab, RoozbehLiu, Tzong-shiAbhyankar, VinayLiu, YanAbramovich, HaimLiu, YuxinAcosta-Vélez, GiovannyLivermore, CarolAdamu-Lema, FikruLo, Kuang YaoAdhikari, RajdeepLobino, MirkoAfferrante, LucianoLodewijks, KristofAgah, MasoudLombardo, SalvatoreAgarwal, AnimeshLoosli, FelixAgarwal, PranayLópez de Lacalle, Luis NorbertoAggogeri, FrancescoLópez, Ana J.Agha, ImadLópez, Miguel ÁngelAhadian, SamadLorusso, MassimoAhmadivand, ArashLou, ShuaiAhmed, DanielLovchik, RobertAhmed, RiazLu, Chia-JungAhmmed, K.M. TanvirLu, FeiAhn, Sung HoonLu, JianAinla, AlarLu, MengAkimoto, AyaLu, RuochenAkram, Muhammad NadeemLu, XiyuanAl Sarawi, SaidLu, YangAl Siyabi, IdrisLu, YaoAl-Ajrash, Saja M. NabatLucia, RomanoAlam, Arif UlLucklum, FriederAlapan, YunusŁuczak, SergiuszAl-Bataineh, SameerLumentut, Mikail F.Aleixandre, ManuelLuo, NingqiAlessio, VernaLuo, QuantianAlexandersen, JoeLuo, Win-jetAlfano, BrigidaLvova, LarisaAlgorri, José FranciscoLyu, HongmingAlhnan, MohamedMaeda, TomokiAli, AzaharMaesato, MitsuhikoAli, Md AzaharMagna, GabrieleAlibakhshikenari, MohammadMahata, Manoj KumarAlistar, MirelaMaher, HassanAlluri, Nagamalleswara RaoMahmood, Md SohelAlmeida, JoseMahy, JulienÁlvarez, MarMaier, Guenther A.Alvaro, D. LatorreMaikap, SiddheswarAlves, Manuel A.Mair, Lamar O.Amama, Placidus B.Maita, FrancescoAmbu, RitaMajewski, LeszekAmini, HamidMajor, IanAmirjani, AmirmostafaMakino, HisaoAmit, IddoMalecha, KarolAmselem, GabrielMalgaretti, PaoloAmza, CatalinMalinge, Jean-MarcAn, SangminMalka, DrorAndres, JuanMandal, Krishna C.Andrikopoulos, GeorgeMandal, SoumyajitAndriotis, OrestisManera, Maria GraziaAngelova, AngelinaMani, Ganesh KumarAral, MustafaMani, VeerappanAramesh, MortezaMantooth, AlanAraromi, OluwaseunMao, WenbinARAUJO, EVANDOMaoz, BenArgurio, PietroMarasso, Simone LuigiAricò, PietroMarc, CourteAriga, KatsuhikoMarcelli, RomoloAristovich, Kirill Y.Marek, GancarzArrabito, GiuseppeMariani, StefanoArumugam, PrabhuMarin, EmmanuelAssefzadeh, MahdiMarkopoulos, AngelosAssender, HazelMarkov, DmitryAtaman, ÇağlarMarkovic, TomislavAvanaki, Mohammad R. N.Marletta, VincenzoAvci, EbubekirMaroufi, MohammadAversa, LucreziaMarques, MarcoAzizi, EbrahimMarquette, Christophe A.Bae, HyungdaeMartel-Foley, JosephBaek, Chang-WookMartín Badosa, EstelaBahreyni, BehraadMartins, RodrigoBai, JingMaruccio, GiuseppeBaier, TobiasMasato, DavideBaigmohammadi, MohammadrezaMassai, DianaBakri-Kassem, MaherMastrangeli, MassimoBaldas, LucienMateo, ReyesBalsamy Kamaraj, AbishekMathews, ScottBamiedakis, NikosMathijssen, Arnold J. T. M.Banan Sadeghian, RaminMatikonda, SiddharthBanerjee, AshisMatsunaga, DaikiBañón, FermínMauri, RobertoBao, YinyinMauriz, ElbaBarbetta, AndreaMažeika, DaliusBarettin, DanieleMazutis, LinasBarille, RegisMcCarty, Owen J.T.Barros, Michael TaynnanMcHugh, KevinBarrot, ChristineMd Foisal, Abu RiduanBarshtein, GregoryMeacci, ValentinoBartlett, Michael D.Meagher, RobertBaskoutas, SotiriosMedina-Sánchez, MarianaBastola, EbinMeen, Teen-HangBastos, JulioMegalini, LudovicoBasu, AnirbanMehdipour, ImanBatmunkh, MunkhbayarMelianas, ArmantasBaù, MarcoMelvin, AdamBauer, A. J.Meneguzzo, FrancescoBauer, RalfMeng, FanbenBazylińska, UrszulaMeng, MaozhouBechelany, MikhaelMeng, XiangchaoBehbahani, Amir HosseinMenzel, StephanBehboodi-Sadabad, FaridMercer, JohnBeilliard, YannMercorelli, PaoloBelfiore, Nicola PioMerenda, AndreaBelharet, KarimMerlo, SabinaBelhocine, AliMerminod, BertrandBelić, DomagojMerrin, JackBella, FedericoMetcalf, Andrew R.Bellantone, VincenzoMiah, Abdul HalimBellos, EvangelosMichael, AronBeltran, Ana M.Michail, ChristosBendahan, Dr. MarcMichel, Carlos R.Benetti, MassimilianoMicic, MiodragBeriáin, AndoniMiguel-Ramos, Mario DeBernard, YvesMiki, NorihisaBernussi, AyrtonMiller, JimmieBertocci, FrancescoMillithaler, Jean-FrançoisBertsch, ArnaudMiloh, TouviaBezryadina, AnnaMin, Jun-HongBhalla, NikhilMin, RuiBhatia, DivijMinakshi, ManickamBhattacharyya, DebabrataMinamiki, TsukuruBhounsule, Pranav A.Minas, GraçaBirch, ChristopherMindaugas, GedvilasBisoyi, Hari KrishnaMinh, Le VanBlanche, Pierre-alexandreMinzioni, PaoloBlázquez-Castro, AlfonsoMiranda, João M.Bockowski, MichalMiranda, João MárioBoda, Sunil KumarMiroshnichenko, AndreyBoddaert, XavierMirzavand, RashidBoeffel, ChristineMishra, NeerajBöhnert, TimMissinne, JeroenBoiger, RomanaMitraki, AnnaBoisseau, SebastienMitsuhiro, TerakawaBolivar, JuanMittal, NiteshBombelli, PaoloMiyamoto, TomoyukiBordeleau, FrançoisMiyazaki, Hideki T.Borges Sebastião, IsraelMobed-Miremadi, MaryamBorovinskaya, Ekaterina S.Modica, FrancescoBorras, AnaMoghimi, MohammadBose, SaurabhMogi, KatsuoBou, Santiago FerrandizMohammad, MahdaviBourillot, EricMohsen, Marani BarzaniBourouina, TarikMojumder, SatyajitBradley, JonathanMokarian, ParvanehBragheri, FrancescaMondal, SudipBreger, JoyceMonsivais-Huertero, AlejandroBreunig, Hans GeorgMontalenti, FrancescoBrisk, PhilipMoon, Byeong-UiBrites, Carlos DSMoon, HyejinBrooks, HadleyMoon, Kyoung-SeokBrouzes, EricMoreira Teixeira, LilianaBrouzgou, AngelikiMori, KotaroBruckbauer, JochenMorley, Nicola ABrunet, PhilippeMorthier, GeertBrzozowska, EwaMosadegh, BobakBuckwell, MarkMoschou, DespinaBujnowski, AdamMoseke, ClausBumah, VioletMouralova, KaterinaBurgués, JavierMouza, Aikaterini A.Burugupally, Sindhu PreethamMoya, Antonio A.Bustillo, JulienMu, XuanCaballero, DavidMuguruma, HitoshiCabanettes, FrédéricMuhammad-Sukki, FirdausCadatal-Raduban, MarilouMüller, GerhardCadore, Alisson RMulloni, VivianaCakmakyapan, SemihMuniyan, SakthivelCalà Lesina, AntoninoMurase, NorioCalahorra, YonatanMurfee, Walter LeeCalaon, MatteoMurmura, Maria AnnaCalaza, CarlosMurshid, NimerCaldas, PauloMuszyński, TomaszCalleja, MontserratMyers, OliverCama, JehangirMyong, Rho ShinCameron, JosephNadagouda, MallikarjunaCampatelli, GianniNadolny, KrzysztofCampo-Deaño, LauraNaeem, UmairCampos, ManuelNaghdi, SamiraCamuñas-Mesa, LuisNaito, ToyohiroCanet Ferrer, JosepNajarian, SiamakCanet-Ferrer, JosepNakagawa, NobuyoshiCanfield, BrianNakamoto, HiroyukiCanfield, ScottNakamura, MasanoriCantero Guisández, José LuisNakano, MichihikoCao, CuongNam, SoojiCao, JiangNama, NiteshCapaldo, PietroNaranjo-Hernández, DavidCaravelli, FrancescoNarayan, Ramasubramanian LakshmiCarminati, MarcoNarayanan Nair, MayaCasals Terre, JasminaNarciso, JavierCaselli, FedericaNaris, SteryiosCasteleiro-Roca, Jose LuisNasiri, NoushinCatarino, SusanaNaskar, SusmitaCauwe, MaartenNawarathna, DharmakeerthiCazzanelli, MassimoNazemifard, NedaCecconi, CiroNee, Matthew J.Celano, UmbertoNejad, Saman NazariCelis, Jean-PierreNgo, Ha DuongCeylan, HakanNguyen, Long QuangChamakos, NikolaosNguyen, ThaiCHAN, Ho YinNguyen, Thanh D.Chan, Hon FaiNguyen, TrieuChan, PakNguyen, Tuan-KhoaChandrahalim, HengkyNguyen-Van, TrietChandrasekaran, ArvindNi, GuangxinChang, Ching-HsinNie, MengyanChang, JiyoungNiesłony, PiotrChang, Liann-BeNightingale, AdrianChang, Sheng-PoNilghaz, AzadehChang, WonkeunNima, Zeid A.Chang, Yao-FengNiu, RanChang, Yuan-JenNivas, Jijil JJChasalevris, AthanasiosNivedita, NiveditaChatare, VijayNiwano, MichioChaudhary, AnkitNoguera, Maria GuixChen, Chang-ChengNose, ToshiakiChen, ChaoyangNosrati, RezaChen, ChiNovak, RichardChen, Chia-YunNozariasbmarz, AminChen, Chien-FuNunes, JanineChen, ChihchenNurzaman, SuryaChen, Chin-ShanO’Mullane, AnthonyChen, HepingOchi, MasayukiChen, JiahaoOgawa, ShinpeiChen, Jian-ZhangOh, Je HoonChen, JinjuOh, Jin YoungChen, Jr-TaiOhlckers, PerChen, JunOjeda, LauroChen, Jyh-JianOk, Jong G.Chen, LiangO’Keefe, KyleChen, LibenOldham, KennChen, Lung-ChienO’Leary, RichardChen, MuyuanOliaei Motlagh, Seyyedeh AzarChen, NanguangOliva, AbelChen, Pang-ShiuOliveira, Thiago E. Alves DeChen, PengyuOlmos, DaniaChen, Pin-ChuanOlszewski, ZbigniewCHEN, Po-YenO’Mahony, ConorChen, QiushuOnoe, HiroakiChen, ShuoOnuki, TeppeiChen, Tao-HsingOrbulov, ImreChen, Wei-TingOrobtchouk, RegisChen, XiangzhongOrsini, AndreaChen, YijiaOrtega-Casanova, JoaquínChen, Yu-ChihOsipov, DmitryChen, Yung-YuOsório, Jonas HChen, YunhanOstadhassan, MehdiCheneler, DavidOu, JianzhenCheng, ChengOu, Jun-YuCheng, Chiang-HoOu, YiyuCheng, KaiOusati Ashtiani, AlirezaCheng, Wood-hiOuyang, XiaolongCheng, ZhenzhouPadilla, José LuisChew, Zheng JunPadmanabhan, JagannathChiadò, AlessandroPaek, JungwookChiang, Ming-HsiPagliara, StefanoChin, Jit KaiPaiè, PetraChinappi, MauroPak, On ShunChiriacò, Maria SerenaPalestri, PierpaoloCho, ChungyeonPallaoro, AlessiaCho, Seong YunPalma, AlbertoCho, Young TaePan, ChengfengCho, Young-hakPancrazio, JosephChocyk, DariuszPandey, SantoshChoi, HeejooPandraud, GregoryChoi, Jane RuPannico, MariannaChoi, JungilPantano, MariaChoi, JungwookPanwar, NishthaChoi, Seung-bokPaoletti, PaoloChoi, SungyoungPapageorgiou, Dimitrios P.Choi, WooSeokPapalou, AngelikiChoi, Woo-SeokPapathanasiou, Athanasios G.Choi, Woo-YoungPappas, DimitriChoiński, DariuszParedes-Madrid, LeonelChou, Jung ChuanParekh, DishitChow, Hwang-CherngParenti, PaoloChow, Kwok-FanPareschi, FabioChristie, SteveParida, KaushikChun, HongguParigger, ChristianCicek, Paul-VaheParis, JuanCioncolini, AndreaPark, Byung-WookClement, MartaPark, ByungwoonClement, YuenPark, Chan HoCochran, SandyPark, ChibeomCocovi Solberg, David JaimePark, Dong HyukCoelho, JoãoPark, Hyeon-CheolCollins, David J.Park, Je-KyunConstantinou, IordaniaPark, JongchanContessi Negrini, NicolaPark, JunyongConway, Daniel E.Park, Kyoung YoulConzuelo, FelipePark, MinCooksey, Gregory A.Park, Min HyukCoppola, SaraPark, SukhoCorrea, DavidPark, SunggookCoskun, M. BulutPark, WookCosta, TiagoPark, Young WookCostantini, MarcoParra, Andrés ConcaCosta-Rama, EstefaníaPassilly, NicolasCourtenay, AaronPastore, RobertoCoutinho, Paulo José GomesPatanè, SalvatoreCovas, José AntónioPatel, RajankumarCox, DavidPatel, SaurinCox, TimothyPatelli, AlessandroCoy, EmersonPathakoti, KavithaCrane, NathanPatrick, ErinCroce, Anna CletaPattanaik, HimansuCrupi, GiovanniPatterson, JenniferCui, BoPaul, Titan C.Czaja, ZbigniewPaun, Maria-AlexandraDa Silva, Jaime Pedro OliveiraPawlak, MichałDagdeviren, CananPeiner, ErwinDai, Ching-LiangPeláez-Moreno, CarmenDai, JingPelle, Flavio DellaDamadam, MohsenPeng, ChangDanovi, DavidePeng, QingDaruwalla, AnoshPennazza, GiorgioDarvishian, AliPensabene, VirginiaDas, PradipPereira, Luiz Fernando RibeiroDas, SambeetaPerenzoni, MatteoDash, BirajaPersaud, KrishnaDau, Van ThanhPersson, Karl-MagnusDavaji, BenyaminPervej Jahan, MuhammadDavid, AdrianPetruska, AndrewDavis, NatePetti, DanielaDavis, PaulPezzimenti, FortunatoDavis, StevePhan, Duc Thanh ThienDawes, JudithPhan, Hoang-PhuongDe Boer, Maarten P.Philipose, UshaDe Ceglia, DomenicoPhilipsen, HaroldDe Gracia, PabloPhillip, JudeDe Lacalle, Luis N. LópezPhillips, Jamie D.De Marco, CarmelaPhuah, Xin LiDe Santi, CarloPicco, LorenDe Santis, FelicePiechna, JanuszDearnley, P. A.Pieri, FrancescoDefay, EmmanuelPierre, Ryan St.Defforge, ThomasPinalli, RobertaDegennaro, LeonardoPiñero, JoseDei, MichelePinho, DianaDel Giudice, FrancescoPini, ValerioDelaporte, PhilippePio Belfiore, NicolaDelattre, CédricPiotto, MassimoDelivopoulos, EvangelosPiro, BenoitDel-Rio Celestino, MercedesPiskin, SenolDelville, Jean-PierrePoenar, DanielD’Emilia, GiulioPolimeni, AntonioDeng, Nan-NanPoot, MennoDeo Malviya, KirtimanPopescu, Mihail N.Dhakal, RajendraPosada-Quintero, HugoDhaundiyal, AlokPostigo, Pablo AitorDi Bartolomeo, AntonioPoumellec, B.Di Caprio, GiuseppePozzi, PaoloDi Flumeri, GianlucaPrabaswara, AdityaDi Pietrantonio, FabioPradhan, NiharDi Sieno, LauraPradhan, ShantanuDicaire, IsabellePrahl Wittberg, LisaDickert, Franz L.Prakash Kottapalli, Ajay GiriDidar, TohidPrakash, ShauryaDijksman, J.F.Prashant, KumarDillert, RalfPrat, MariaDilonardo, ElenaPrelipceanu, MariusDimitrakellis, PanagiotisPriante, DavideDimitrakis, PanagiotisPrieto-Simon, BeatrizDing, SonglinPriye, AashishDing, XiaoyunProietti, EmanuelaDinh, ToanProust, JulienDo, Thanh NhoPtaszek, MarcinDochshanov, AldenPu, HaihuiDoeven, EganPuchades, IvanDonaldson, NickPuglisi, AlessandraDong, LixinPullano, SalvatoreDong, RenhaoQamar, AfzaalDoostmohammadi, AminQian, JunDowling, KarenQian, ShizhiDrese, Klaus StefanQian, ZhiyuanDu, KeQin, YihengDuan, GuangwuQiu, TianDuan, XiyuQiu, ZhenDuarte Campos, Daniela FilipaQuan, ZhongyiDuarte Marafona, JoséQuero, GiuseppeDubey, NileshkumarQuintana, IbanDubey, RichaRadhakrishna, UjwalDulf, Eva H.Radtke, AleksandraDumitru, Andra-CristinaRadtke, MariuszDumur, FrédéricRaeisHosseini, NiloufarDutoit, BertrandRaeis-Hosseini, NiloufarDziuda, ŁukaszRahman, M ArifurEbbens, StephenRahman, TanzilurEbrahimi Warkiani, MajidRai, PrakashEbralidze, Iraklii I.Raj, AbhishekEgan, PaulRajaraman, SwaminathanEgan, Paul F.Rajnicek, AnnEhrmann, AndreaRajta, IstvanEickenscheidt, MaxRamakrishnan, SathishEinfeldt, SvenRamos, DanielElahi, HassanRana, Abu Ul Hassan SarwarElbuken, CaglarRanjkesh, AmidElkaseer, AhmedRao, SmithaEl-kharouf, AhmadRaposo, MariaElsayed, MohannadRaptis, IoannisElvira, Katherine S.Rashidi, ArmanEmadi, ArezooRashtian, HoomanEngelbrecht, RainerRasponi, MarcoErb, RandallRathod, VivekErenas, Miguel MaríaRavasio, ChiaraErtl, PeterRavindra, NuggehalliEscobedo, CarlosRebollar, EstherEsfahani Monfared, YasharReboud, JulienEsfandyarpour, RahimRech, IvanEsposito, FlavioReddy Chittamuru, Sai VineelEstellé, PatriceReddy, AmaranathaEsteve-Romero, JosepReece, Lisa M.Ettayapuram Ramaprasad, Azhagiya SingamRega, RominaEwen, JamesReiche, Christopher F.Fain, BrunoReid, Russell C.Faller, Lisa-MarieReig, CàndidFan, XinReinhard, FriedemannFan, XudongRej, SouravFan, ZhongmingRen, LiqiangFang, HuiRen, YukunFang, JianRenggli, KasperFarokhi, HamedRenò, FilippoFassi, IreneRezaei, BabakFateixa, Sara Isabel AugustoRhee, HuinamFeiertag, GregorRhi, Seok-HoFeinaeugle, MatthiasRho, Hoon SukFelgueiras, Helena P.Rho, JunsukFelt, WyattRichardson, RobertFeng, HuichengRijckaert, HannesFeng, PhilipRimoldi, LucaFernandes, FábioRitz, UlrikeFernandez, SusanaRivadeneyra, AlmudenaFerrari, MicheleRizal, ConradFerrás, L. L.Rizzuti, BrunoFerrero Martín, Francisco J.Robert, NewcombFick, JochenRobinson, Jacob T.Firebaugh, SamaraRoccaforte, FabrizioFischer, GeorgRodrigo, Peter JohnFischer, PeerRodrigues, RaquelFodor, Petru S.Rodriguez, NoelFolch, AlbertRoh, Kyung-HoFortmann, CharlesRojas-Laguna, RobertoFortunati, IlariaRoldán Gómez, Juan JesúsFournel, FrankRomani, PatriziaFragouli, DespinaRomano, LuciaFrancais, OlivierRomano, ValerioFrancés-Soriano, LauraRoppolo, IgnazioFrangi, AttilioRoque, AntonioFranssila, SamiRosa, ViniciusFrenea-Robin, MarieRosado, JaimeFreudenberger, JürgenRoth, JohannesFriedt, Jean-MichelRothbauer, MarioFrogley, MarkRozema, JosFujii, FumitakeRóżycki, BartoszFukuda, TakeshiRubino, EdoardoFunari, RiccardoRubio, Leire RuizFurutani, KatsushiRuggeri, SerenaFuso, FrancescoRuiz-Díez, VíctorFutai, NobuyukiRusu, CristinaG. Baboly, Mohammad HoseinRydosz, ArturGain, Asit KumarRyger, IvanGaitas, AngeloRyou, Jae-HyunGala, Rikhav PRyu Cho, Yu KyoungGalfetti, LucianoRyu, Han-YoulGalinetto, PietroSafaei, AlirezaGallis, SpyrosSaffari Pour, MohsenGanesana, MallikarjunaraoSaggiomo, VittorioGanser, ChristianSahoo, DeepakGao, AnmingSahore, VishalGao, HaoSaijo, YoshifumiGao, NanSailhan, FrancoiseGao, YujiSainidou, RebeccaGarcia-Gonzales, CarlosSaitou, MasatoshiGarcía-Lastra, Juan MaríaSajid, MuhammadGarcía-Sánchez, PabloSakai, MasatoshiGardeniers, HanSakharova, NataliyaGarrill, AshleySalari, AlinaghiGartia, Manas RanjanSalem, BassemGastaldo, PaoloŠalić, AnitaGautam, SiddharthSalinas, Dídac GómezGautier, GaelSalmon, HugoGebicki, JacekSalvador, DueñasGeiser, Martial H.Salvatore, SurdoGendron, PaulSamal, MonicaGeorgiev, YordanSamal, SangramGeorgopanos, ProkopiosSameoto, DanGerardino, AnnamariaSamiei, EhsanGerbeth, GunterSan Miguel, AdrianaGhafar-Zadeh, EbrahimSánchez Egea, AntonioGhanmi, HelmiSanchez, Jean-BaptisteGhannad-Rezaie, MostafaSanchez-Martinez, Juan JoséGhantasala, MuralidharSanchez-Rojas, Jose LuisGhatkesar, Murali KrishnaSánchez-Soto, Luis L.GHAYESH, MERGENSankaran, Kamatchi JothiramalingamGhayesh, Mergen H.Sant, HimanshuGhidelli, MatteoSant, SaurabhGhisi, AldoSantiago, JuanGhosh, ArijitSaravanan, T JothiGiasin, KhaledSaremi, MehdiGibiino, Gian PieroSarkar, DhrubaGieseler, JanSarma, RaktimGijsenbergh, PieterSarris, Ioannis E.Gili, Valerio FlavioSattari, HamedGiltinan, JoshuaSaucedo Espinosa, Mario AlbertoGimsa, JanScaini, DenisGlüsen, AndreasSchiavone, GiuseppeGnyba, MarcinSchintke, SilviaGoga, NicolaeSchirhagl, RomanaGokhale, Vikrant JayantSchlappi, TravisGolub, Victor V.Schmedake, Thomas A.Gomes, HenriqueSchmitt, KatrinGómez-Espinosa, AlfonsoSchneider, MichaelGómez-Pastora, JeniferSchönauer-Kamin, DanielaGong, MaogangSchönthal, Axel H.Gong, MaxSchulz, Volker PaulGong, ShuSchwab, ChristianGong, ZhixiongScirè, SalvatoreGonzález, HernánScorza, AndreaGonzález-Romero, ElisaScotognella, FrancescoGordeev, SergeyScotti, GianmarioGraaf, Ger DeSébastien, SartGräbner, DanielSegreto, TizianaGrabowski, MarcinSegura, JaumeGrange, RachelSeibel, ArthurGrasso, SalvatoreSeichepine, FlorentGrattieri, MatteoSelvaganapathy, P. RaviGrau, GerdSemmar, NadjibGray, Michael D.Seok, OgyunGreener, JesseSeok, SenhoGregáň, JurajSeol, Myeong-LokGrenci, GianlucaSeredyński, MirosławGric, TatjanaSerpooshan, VahidGrieve, James A.Serry, MohamedGrigsby, Christopher L.Sethmann, IngoGrillo, FedericoSetti, DineshGrisé, FabienSevcsik, EvaGrivel, Jean-ClaudeShafique, MuhammadGrossi, MarcoShah, SahilGrunwald, RuedigerShakeel, HamzaGu, HaichangShalaby, MohamedGu, WentingShalev, GilGuan, XiaoweiShang, LuoranGueorguiev, GueorguiShanmugam, VinodhGuerra Rosa, LuisShao, LeiGuijt, RosanneShao, PengGuiney, Linda M.Sharker, Shazid Md.Gulari, ErdoganSharma, DeepikaGuldiken, RasimSharma, DeeptiGulinatti, AngeloSharma, MrigankGulizzi, VincenzoSharma, PradipGullo, MaurizioShavezipur, Mohammad K.Gumfekar, SarangShaygan, MehrdadGumuscu, BurcuSheard, SteveGuo, JianglongShehata, NaderGuo, PingShen, Chih-HsiungGuo, QingboSheri, MadhuGuo, QiuquanSherwood, Joseph M.Guo, ShuangzhuangShi, ChengzhiGupta, VipulShi, WenGut, KazimierzShields IV, C. WyattGutierrez, Ana MariaShields, Charles WyattGutiérrez-Capitán, ManuelShields, PhilipGutruf, PhilippShields, WyattHaddadi, AbbasShih, SteveHaffner, ChristianShim, JiwookHage-Ali, SamiShimada, KeitaHagelauer, AmelieShin, Bo-SungHagen, Christopher L.Shin, SangJoonHajizadegan, MehdiShirahata, NaotoHamelin, BenoitShivakumar, Vibhav BharadwajHamer, PhilipShoji, KanHammarström, BjörnShpaisman, HagayHamon, MorganShrestha, BhanuHan, Gill SangShrirao, AnilHan, Jin-WooShtepliuk, IvanHan, YiweiShum, HenryHane, KazuhiroSiciliani, MarioHao, GuangboSidorenko, AlexanderHarada, YukihiroSieben, VincentHarnois, MaximeSier, CornelisHarris, Andrew R.Signore, GiovanniHashimoto, MasahikoSilva, Francisco J. G.Hassanin, HanySilva, SusanaHastings, ToddSim, Gi-DongHatta, AkimitsuSimanjuntak, Firman MangasaHaubner, RolandSimmchen, JulianeHaun, JeredSimões, SóniaHawash, ZaferSingh, Ajay VikramHayakawa, MasayukiSingh, JaiHayakawa, TakeshiSingh, VeenaHe, DongfengSkardal, AleksanderHe, LingnaSlouka, ZdenekHe, MeiSmall, AlexHe, WeiSmith, MichaelHeinemann, RobertSo, HongyunHejazian, MajidSo, Kwok KanHellenkamp, Bjoern DavidSoares, Ruben R.G.Hemmatifar, AliSobotta, ŁukaszHernando-Garcia, JorgeSohn, Dong KeeHerrnsdorf, JohannesSohn, Jung WooHerth, EtienneSohrabi, SalmanHeydari Gharahcheshmeh, MeysamSomà, AurelioHida, HirotakaSong, Hyun-CheolHidalgo López, José AntonioSong, JonathanHijikata, YasutoSong, QinghuaHilt, OliverSong, ShigengHirai, YoshikazuSong, Yong-Ak (Rafael)Hirano, HidekiSong, Young MinHirtz, MichaelSontakke, Atul D.Hjelme, Dag RoarSoomro, Toufique AhmedHo, DanielSorrentino, AndreaHo, Jeng-RongSortino, MarcoHo, Wei-YuSosnowski, Tomasz R. Hoath, StephenSoto Rodriguez, Paul Eduardo DavidHoettges, KaiSrivastava, SaurabhHomola, TomášStahl, FrankHong, ChungpyoStanley, ClaireHong, MinghuiStano, PasqualeHong, Suck WonStavrakis, StavrosHong, SukjoonSteager, EdwardHorsfall, AltonStephen, Neil G.Hsiao, Chun-ChingSternberg, AndrewHsiao, Fu-yuenSthal, FabriceHsiao, H.L.Stiharu, IonHsiao, Vincent K.S.Stoop, Ralph L.Hsieh, KuangwenStornelli, VincenzoHsin, Yue-MingStrakosas, XenofonHsu, Huan-HsuanStrehlitz, BeateHsu, Jin-ChenStrelniker, YakovHsu, Wei-LunStride, John ArronHsue, Albert Wen-JengStringer, Jonathan EdwardHu, ChunxiaoStruk, PrzemyslawHu, MiaoStuart, Marc C. A.Hu, Wei-FanStumpe, JoachimHuang, Cheng-MingSu, HangHuang, Chia-HuaSu, Pi-GueyHuang, Chih-HsienSubbaraman, HarishHuang, Chi-HsienSuder, WojciechHuang, Hong-YuanSullivan, JohnHuang, Kuo-ChengSullivan, TimothyHuang, Po-HsunSun, ChenHuang, QijinSun, GongchenHuang, Shyh-ChourSun, JianHuang, SunanSun, JiningHuang, Tzu-YenSun, KeyeHuang, YingSun, Kien-WenHuang, Yu-HsiSun, MengHuang, Zhaoran (rena)Sun, XiankaiHughes, MichaelSun, Yung-ShinHuguet, Silvia SoriaSung, JaeyongHummon, MatthewSunna, AnwarHung, Shao-KangSurace, RossellaHussain, GhulamSutlief, ArinHuynh, Thanh-CanhSuzuki, KenichiroHwang, Dong-HwanSuzuki, TakaakiHwang, Suk-WonSwami, NathanHwang, YongsungSwiderek, PetraHyun, Kyung-AŚwiercz, RafałIbarlucea, BergoiSyms, RichardIbrahim, AliSzczęsny, SzymonIbsen, StuartSzukalski, AdamIchikawa, YoTabassum, ShawanaIliopoulos, KonstantinosTaboryski, RafaelIm, HyungsoonTaboryski, Rafael J.Ino, KosukeTadrist, LoïcIonescu, Elena RodicaTahara, HironobuIrisawa, ToshifumiTait, NiallIsa, FabioTakahashi, YasuoIshida, TadashiTakamatsu, SeiichiIsozaki, AkihiroTalaat, AhmedIstrate, DanTalò, GiuseppeIwami, KentaroTan, HuaJackson, MarkTan, Lling LlingJackson, NathanTan, Rex XiaoJadidi, MehdiTang, GongyueJadwicienczak, WojciechTang, ShiyangJafferis, Noah T.Tang, XinJagdheesh, R.Tani, HirofumiJähn, KatharinaTao, KaiJakieła, SławomirTao, LiJakubik, Wiesław P.Taran, ElenaJames, SagilTatar, ErdincJamshidi, ReihanehTate, DanielJanas, DawidTawfik, Hani H.Jang, HanminTeghil, RobertoJang, Ho WonTejedor, José Antonio GómezJang, Jae-WonTekes, CoskunJanissen, RichardTelichko, ArseniiJastrzębska, ElżbietaTemiz, YukselJen, Chun-PingTheres Baby, TessyJeong, Ok ChanThorsen, ToddJeong, Yong JinThurgood, PeterJha, Abhinav K.Tian, WeisongJi, LiTien, Wei-HsinJia, YuTittmann, Bernhard RainerJiang, BaoyangToda, MasayaJiang, YouhuaTodea, AnamariaJin, ChenyuToh, Yi-ChinJin, QianruTomkowski, RobertJin, XiaoliangTonacci, AlessandroJing, WenwenTonello, AlessandroJing, WumingTorisawa, YusukeJohansson, ChristerToroń, BartłomiejJönsson-Niedziolka, MartinTorres-Diaz, IsaacJOSHI, NIRAVKUMARTran, Duy PhuJuan-Colás, JoséTropea, CameronJuang, Yi-JeTrotta, GianlucaJugessur, AjuTrovato, FabioJun, KondohTruong, Vi-KhanhJung, DaewoongTrusiak, MaciejKabir, K. M. MohibulTruskey, George A.Kacem, NajibTsai, Ang-ChenKainz, AndreasTsai, Chia-Hung DylanKaji, NoritadaTsao, Chung ChenKalantar-zadeh, KouroshTsao, Chung-chenKa-Meng, LeiTseng, Sheng-HsiangKameoka, JunTseng, Zong-LiangKaminski, TomaszTsiigkaridis, John (Ioannis)Kamm, Roger DaleTsow, Francis (Tsing)Kamperman, TomTsubota, Ken-ichiKämpfer-Homsy, AlexandraTsuchiya, YoshishigeKanda, KensukeTsuchiya, YouichiKang, BongchulTsuji, TetsuroKang, ChanKyuTsukamoto, TakashiroKang, Dong-KuTsvetkov, NikolaiKang, Dong-WonTu, ChengKang, Joo H.Turkmen, MustafaKang, SaewonTyaginov, StanislavKang, Seok-WonUchida, SatoshiKang, SeungYeonUeta, IkuoKang, Sung-MinUm, Doo-seungKang, Tae GonUrban, Matthew W.Kang, Yang JunUrbano, Ana MargaridaKanik, MehmetVafapour, ZohrehKannappan, RamaswamyVagionas, ChristosKano, ShinyaVaikuntanathan, VisakhKantak, ChaitanyaValiente, DavidKao, Tsung ShengValov, IliaKaplun, DmitriiValsaraj, AmithrajKaps, SörenValvano, StefanoKarakasidis, TheodorosVan Dam, R. MichaelKarande, RohanVan De Put, MaartenKashaninejad, Navidvan der Meer, Andries D.Kashimura, KeiichiroVan Der Sluis, OlafKasiteropoulou, DoraVan Doorn, Jaap.Kassavetis, SpyrosVan Toan, NguyenKathuria, HimanshuVannini, GiorgioKato, MasashiVassant, SimonKaushik, AjeetVattikuti, Surya Veerendra PrabhakarKawahara, TomohiroVázquez, LuisKawashima, KenjiVazquez, Rebeca MartinezKeating, AdrianVeerasikku Gopal, DeepaganKechagias, John D.Vegendla, PrasadKeleher, Jason J.Velasco-Vélez, Juan JesúsKern, Thorsten A.Velazquez-Perez, Jesus EKeskinbora, KahramanVenediktov, VladimirKeszler, AgnesVenkatasubramanian, AnandramKeszöcze, OliverVenkatesh, Alagarswamy G.Ketkaew, JittisaVerona, EnricoKhafaji, SalwanVerotti, MatteoKhan, MuhammadVespini, VeronicaKhang, D. Y.Viefhues, MartinaKhanna, SouravViespe, CristianKhansur, Neamul HayetVijayavenkataraman, SanjairajKho, Hong-SeopVila-Comamala, JoanKhodadadi, MohammadVilian, A.T. EzhilKhoo, Bee LuanVincenti, M. A.Khoshmanesh, KhashayarVisvanathan, RayshanKiani, AmirkianooshVitelli, MassimoKim, AlbertVitry, PaulineKim, Brian N.Vocale, PamelaKim, Byeong HeeVoglhuber-Brunnmaier, ThomasKim, Dal HyungVohnsen, BrianKim, Dong-HweeVoiculescu, IoanaKim, Heung-KyuVolgin, Vladimir M.KIM, Heuy DongVollbrecht, JoachimKim, Hong NamW.k., FungKim, HyunseokWaddell, EmanuelKim, JaeyounWagner, NormanKim, JanghoWakai, FumihiroKim, JeonghyunWalczak, RafałKim, Jin SikWalewyns, ThomasKim, JinhoWaller, Erik HagenKim, JinwookWang, BaomingKim, Jung-SikWang, CemingKim, KwanohWang, ChunjinKim, KyobumWang, Dung-AnKim, Prof. Dr. ChoongikWang, HaoKim, Sang MoonWang, HongboKim, Sang-JaeWang, Hsiang-ChenKim, SangkilWang, Hung-YuKim, SangwanWang, LihuaKim, SeokminWang, LingKim, SoaramWang, Michael CaiKim, SongkilWang, MingkangKim, SooyeonWang, MinqiangKim, Sung JaeWang, PengKim, SungjunWang, PengtaoKim, SunjunWang, QiuguKim, Wook-BaeWang, Shau-ChunKim, Young LaeWang, TaoKimura, MutsumiWang, WendongKinahan, David JWang, Xiaoguang (William)Kirchner, RobertWang, XiaosongKirillova, AlinaWang, YingKityk, IwanWang, Yu-LinKlapez, MartinWang, YulingKlein, SusanneWang, ZhenfengKlini, ArgyroWang, ZhijianKlit, PederWang, ZhipingKnapkiewicz, PawełWang, Zhong LinKnospe, Carl R.Wang, ZhongruiKobayashi, MakikoWang, ZhuqingKoens, LyndonWang, ZuankaiKoh, Kai SengWatanabe, HiromichiKõiva, RistoWatanabe, YuichiroKokkinos, ChristosWatson, ScottKong, CharlieWebster, EricKong, Xiang-TianWegener, JoachimKönözsy, LászlóWei, ChengliKonstantaki, MariaWei, QingshanKontziampasis, DimitriosWeigel, RobertKoo, ChiwanWeis, ChristianKopra, KariWeller, Michael G.Korampally, VenumadhavWells, LauraKosar, AliWen, HaoranKoutselas, IoannisWeng, Yung-JinKovalev, AlexanderWesterberg, Lars-GöranKoyano, TomohiroWesterhausen, ChristophKulinich, SergeiWhyte, GraemeKulshreshtha, PrashantWibowo, DavidKumar, AnilWiecha, Peter R.Kumar, HarishWiegerink, RemcoKumar, PankajWiklund, MartinKumar, SatishWilkins, Richard TKumar, SuhasWilksch, PhilipKumar, VivekWillert, AndreasKumavor, Patrick D.Williams, JohnKung, Yu-ChunWilliams, Noelle S.Kunihisa, TashiroWilliams, Stuart J.Kuo, Wen-chengWilson, DanKuramoto, MasaruWiniarski, TomaszKurlyandskaya, Galina V.Wiśniewski, RemigiuszKurosawa, OsamuWitkowska Nery, EmiliaKusters, IljaWitte, HartmutKwok, Ngai MingWlodkowic, DonaldKwon, BeomjinWojciechowski, SzymonKwon, Jae SungWojkiewicz, Jean-LucKwon, Jae-SungWojnicki, MarekKwon, Kye-SiWolszczak, PiotrKwon, TaehongWong, Lai MunKwon, WoosungWong, PakKyratsis, PanagiotisWu, ChenshengLabuta, JanWu, JiandongLackovic, IgorWu, KaiLaforge, François O.Wu, NanLafratta, ChristopherWu, Shih-JehLagarde, FlorenceWu, Tian-LiLaguarda-Miró, NicolásWu, XiaofengLahoz, RuthWu, XinLai, Chien-ChihWu, XuejianLai, Ngoc DiepWuethrich, AlainLai, Pin-KuangWujcik, Evan K.Lam, RaymondXianyu, YunleiLanders, JamesXiao, BoqiLang, WalterXiao, LiLanger, Jerzy JXiao, YuanLanotte, LucaXie, XiaopengLardies, JosephXie, YuliangLautner, GergelyXiong, JiaqingLavrentovich, OlegXu, ChunfuLaw, Cheryl SuwenXu, LuLazarus, NathanXu, YongLe Borgne, BriceYaakoubi, NourdinLe Febvrier, ArnaudYadav, HemrajLe Moal, PatriceYadavali, SagarLe, Hoa ThanhYadavalli, Nataraja SekharLeal-Junior, ArnaldoYaïci, WahibaLederer, MartinYaji, KentaroLedo, AnaYalikun, YaxiaerLee, AlbertYamada, IchiroLee, ChanghoYamaguchi, MasakiLee, DaehoYamazaki, HirohitoLee, DongwooYan, YongkeLee, Han EolYang, Chih-TsungLee, Hyun HoYang, GengLee, HyungwooYang, GuangLee, Hyunjoo J.Yang, LiangLee, HyunseopYang, MinyangLee, J. B.Yang, RuiguoLee, JaesungYang, XusanLee, JeonghoonYang, YansongLee, Jeong-SooYang, YunzeLee, JongwonYang, ZongyinLee, JoonheeYano, MitsuakiLee, Ju-HyuckYao, JunLee, KeekeunYapici, Murat KayaLee, MinbaekYazdan-Shahmorad, AzadehLee, Moon GuYeh, Yin-TingLee, Myung-JaeYeo, HongLee, Sang JinYeom, JunghoonLee, SanghoonYi-chin, TohLee, Seok JaeYo, TanakaLee, Sung SikYong, Kar WeyLee, Tae YoonYoon, GiwanLee, Tae-HakYoon, HongJoonLee, WeiYoon, Hyeun JoongLee, Wen-JenYoon, Jae WoongLee, Yin-WenYou, Jae BemLee, Yun-HanYou, Jeong HoLei, UYu, Chin-pingLeissner, TillYu, Feiqiao BrianLenci, StefanoYu, HakkiLeonidas, PalilisYu, Hsing-ChengLeporatti, StefanoYu, Jae WoongLesiak, PiotrYu, KaiyanLettieri, MariateresaYu, Wan ChinLewandowski, WiktorYu, XingeLi, ChongYu, ZiyiLi, DuoYuan, LeiLi, FeiYun, JinHyeonLi, FengZahn, Jeffrey D.Li, HaijunZambelli, CristianLi, HeZareinia, KouroshLi, HuaizhongZavodszky, GaborLi, HuanZega, ValentinaLi, JinxingZenith, FedericoLi, LinZhai, YaoLi, MengZhang, DongshiLi, Ming-HuangZhang, EnxiaLi, PengZhang, GuigenLI, QingfengZhang, HongLi, RuyaZhang, HuilongLi, ShilongZhang, JinjinLi, TaoZhang, NanLi, TianchengZhang, ShimingLi, XiangZhang, TianshengLi, XiaoZhang, WenxuLi, XiaotianZhang, WujieLi, YangminZhang, XianLi, YiZhang, XinquanLi, YifeiZhang, XuLi, YiyanZHANG, YILi, ZidaZhang, YinanLiang, Chi-TeZhang, YongLiao, Ming HanZhang, YunyanLiao, YabinZhang, ZhienLignos, IoannisZhang, ZhouLigrani, PhillipZhao, ChumingLim, Chun YeeZhao, DingLim, JiseokZhao, HubinLim, SoomanZhao, JingzhouLim, Yee YanZhao, WeifengLima, IvanZhao, YanyuLima, Rui A.Zheng, BoLin, Chih-LangZheng, BocongLin, Chih-MingZheng, JinchuanLin, Chih-TingZheng, ShijieLin, FrancisZheng, YiLin, Gong-RuZhou, JiajingLin, JianhanZhou, JianLin, LinhanZhou, RanLin, MengZhou, YufengLin, Shu-YenZhu, HongyangLin, T. HZhu, JunjieLin, TongZhu, ShanLin, YuankunZhu, WanlinLin, Yu-HsienZhu, XiaoliangLin, ZhiyuanZhu, YiboLinß, SebastianZhu, ZijieLinzon, YoavZhuang, XinLionetto, FrancescaZhuo, YueLipiński, SewerynZi, YunlongLiu, BifengZimmerman, WilliamLiu, BoZinno, RaffaeleLiu, Cheng-HsienZolfagharian, AliLiu, Chien-HaoZöllner, Jens-PeterLiu, Chung-ChiunZou, HaiyangLiu, GuozhenZuhlke, Craig A.Liu, JunZuo, Lijian

